# Assessment of Methodological Quality of Economic Evaluations in Belgian Drug Reimbursement Applications

**DOI:** 10.1371/journal.pone.0085411

**Published:** 2013-12-30

**Authors:** Steven Simoens

**Affiliations:** KU Leuven Department of Pharmaceutical and Pharmacological Sciences, Leuven, Belgium; University of Calgary, Canada

## Abstract

**Objectives:**

This paper aims to assess the methodological quality of economic evaluations included in Belgian reimbursement applications for Class 1 drugs.

**Materials and Methods:**

For 19 reimbursement applications submitted during 2011 and Spring 2012, a descriptive analysis assessed the methodological quality of the economic evaluation, evaluated the assessment of that economic evaluation by the Drug Reimbursement Committee and the response to that assessment by the company. Compliance with methodological guidelines issued by the Belgian Healthcare Knowledge Centre was assessed using a detailed checklist of 23 methodological items. The rate of compliance was calculated based on the number of economic evaluations for which the item was applicable.

**Results:**

Economic evaluations tended to comply with guidelines regarding perspective, target population, subgroup analyses, comparator, use of comparative clinical data and final outcome measures, calculation of costs, incremental analysis, discounting and time horizon. However, more attention needs to be paid to the description of limitations of indirect comparisons, the choice of an appropriate analytic technique, the expression of unit costs in values for the current year, the estimation and valuation of outcomes, the presentation of results of sensitivity analyses, and testing the face validity of model inputs and outputs. Also, a large variation was observed in the scope and depth of the quality assessment by the Drug Reimbursement Committee.

**Conclusions:**

Although general guidelines exist, pharmaceutical companies and the Drug Reimbursement Committee would benefit from the existence of a more detailed checklist of methodological items that need to be reported in an economic evaluation.

## Introduction

Similar to many other jurisdictions, an economic evaluation needs to be submitted as part of a drug reimbursement application to the Belgian Drug Reimbursement Committee since 2002 [1]. In Belgium, this requirement holds for so-called Class 1 drugs only, i.e. drugs that exhibit an added therapeutic value over existing alternatives. In this respect, therapeutic value is a broad concept that is defined by law to encompass both the efficacy and effectiveness of a drug, its safety, applicability and user friendliness [2]. The drug reimbursement decision takes into account multiple factors, including the therapeutic value of the drug, its price, the importance of the drug in clinical practice, its budget impact and cost-effectiveness.

In order to inform the design and reporting of economic evaluations in the context of drug reimbursement applications, the Belgian Healthcare Knowledge Centre (Federaal Kenniscentrum voor de Gezondheidszorg (KCE)) issued methodological guidelines in 2008 [3]. This document provided guidance on 11 methodological items relating to the perspective of the economic evaluation, target population, comparators, analytic technique, study design, calculation of costs, estimation and valuation of outcomes, time horizon, modelling, handling of uncertainty and testing for robustness of results, and discounting. The document also proposed a template for reporting the economic evaluation.

Pharmaceutical companies are required to adhere to the KCE methodological guidelines or provide a justification for any deviation from the guidelines. The drug reimbursement application is evaluated by the Drug Reimbursement Committee in the scientific evaluation report, including an assessment of the methodological quality of the economic evaluation. The pharmaceutical company then has the opportunity to respond to any issues raised by the Drug Reimbursement Committee. 

Given that the KCE guidelines purport to increase the methodological quality, transparency and consistency of submitted economic evaluations, it is important to assess whether pharmaceutical companies comply with guidelines and to identify those methodological items where deviations occur. Few studies have examined the methodological quality of economic evaluations in drug reimbursement applications. A Dutch study reviewed pharmaco-economic submissions and found that economic evaluations tended to comply with some methodological items, but were deficient with respect to other items [4]. A Canadian study observed that many economic evaluations used to inform cancer drug reimbursement were flawed, thus pointing to the need to assess the quality of the evidence submitted [5].

The aim of this paper is to assess the methodological quality of economic evaluations included in Belgian reimbursement applications for Class 1 drugs. To this effect, the paper identifies those areas where economic evaluations do not comply with KCE guidelines and provides recommendations for pharmaceutical companies on how to improve the methodological quality of economic evaluations. The paper also comments on the quality of the methodological assessment undertaken by the Belgian Drug Reimbursement Committee itself and formulates recommendations on how to enhance the quality of that assessment.

## Materials and Methods

A descriptive analysis assessed the methodological quality of economic evaluations of 19 Class 1 drugs for which a reimbursement application was submitted to the Belgian Drug Reimbursement Committee. Although orphan drugs are classified as Class 1 drugs in Belgium, orphan drugs were not considered because the reimbursement application does not need to include an economic evaluation.

Given that the KCE guidelines were introduced in 2008 [3] and that there may have been a learning curve in implementing the guidelines, the analysis included all successive reimbursement applications for Class 1 drugs submitted during 2011 and Spring 2012. 

Pharmaceutical companies that submitted a reimbursement application for a Class 1 drug during this time period were contacted with a view to gaining access to the following documents: a) the economic evaluation included in the reimbursement application submitted by the company; b) the assessment of that economic evaluation by the Drug Reimbursement Committee in the scientific evaluation report; and c) the response to that assessment by the company. All contacted pharmaceutical companies agreed for their drug reimbursement application to be included in this study, except for one company (which did not provide a reason for its refusal).

Information about the methodological quality of the economic evaluation was extracted from each reimbursement application using a standardized reporting template. This template broke down the 11 general KCE guidelines [3] into a more detailed checklist of 23 items that are covered in the KCE guidelines (see Table 1). The assessment considered whether economic evaluations comply with guidelines or whether an adequate justification is provided by the company in case of non-compliance. If a methodological item was not relevant to a specific economic evaluation, ‘not applicable’ was recorded in the template. 

**Table 1:** Compliance of economic evaluations in Belgian drug reimbursement applications with KCE methodological guidelines [3].

	*Drug A*	*Drug B*	*Drug C*	*Drug D*	*Drug E*	*Drug F*	*Drug G*	*Drug H*	*Drug I*	*Drug J*	*Drug K*	*Drug L*	*Drug M*	*Drug N*	*Drug O*	*Drug P*	*Drug Q*	*Drug R*	*Drug S*	*Compliance rate*
Economic evaluation is conducted from health care payer perspective	1	1	1	0	1	1	0	1	1	1	1	1	1	0	1	1	1	1	0	79%
																				(15/19)
Patient population of economic evaluation is consistent with population defined in clinical part of reimbursement application	1	1	1	1	1	1	1	1	1	1	1	1	1	1	1	1	1	1	1	100%
																				(19/19)
An appropriate justification is provided for subgroup analyses	1	NA	NA	NA	0	1	NA	NA	1	NA	NA	NA	1	NA	1	NA	NA	NA	NA	83%
																				(5/6)
The comparator is the most relevant alternative treatment for the indication of the drug	1	1	1	1	1	1	0	1	1	1	1	0	0	1	1	0	0	1	1	74%
																				(14/19)
Appropriate justification is provided for indirect comparison and its limitations are described	NA	0	NA	NA	NA	0	NA	NA	NA	NA	NA	0	NA	NA	0	NA	NA	NA	NA	0%
																				(0/4)
CEA is performed based on single, dominant patient-relevant outcome	NA	NA	1	0	0	NA	0	NA	NA	NA	0	NA	NA	NA	NA	0	0	1	0	22%
																				(2/9)
CUA is performed based on multiple patient-relevant outcomes or quality of life	0	1	1	1	1	0	0	1	0	1	1	0	0	0	1	0	0	1	0	47%
																				(9/19)
An incremental cost-effectiveness or cost-utility ratio is calculated	0	1	1	1	1	0	1	1	0	0	0	1	1	1	1	1	1	1	1	74%
																				(14/19)
Economic evaluation is based to some extent on data from RCTs or observational studies comparing drug with comparator	1	1	1	1	1	1	1	1	1	1	1	1	1	1	1	1	1	1	1	100%
																				(19/19)
All relevant costs are identified from health care payer perspective	0	1	1	1	1	1	0	1	1	0	0	1	1	1	1	1	0	1	0	68%
																				(13/19)
Resource use is measured based on observations or literature, and not solely on expert opinion	0	1	1	0	1	1	0	1	1	1	1	1	1	1	1	1	1	1	1	84%
																				(16/19)
Validated sources are used for unit costs	0	1	1	1	1	1	1	1	1	1	1	1	1	1	1	0	1	1	0	84%
																				(16/19)
Unit costs are expressed in values for the current year	0	0	1	1	0	1	0	1	1	1	0	0	0	0	0	0	1	0	0	37%
																				(7/19)
The reference price is used for the comparator when generic drugs exist	NA	NA	1	NA	0	1	0	NA	NA	NA	NA	1	NA	NA	0	1	NA	0	1	56%
																				(5/9)
Final outcome measures are used instead of intermediary outcomes	1	1	1	1	1	1	NA	1	1	1	1	1	1	1	1	1	NA	1	0	94%
																				(16/17)
Life expectancy estimates are based on Belgian age-specific life tables and on all-cause mortality	0	0	0	0	NA	0	NA	0	0	1	0	NA	0	0	NA	0	NA	0	NA	8%
																				(1/13)
Health states are described using a generic instrument and valued by the Belgian general public	0	0	0	0	0	0	NA	0	0	0	0	0	1	0	0	NA	NA	0	NA	7%
																				(1/15)
Time horizon reflects the period over which costs and outcomes of drug and comparator are expected	1	1	1	1	1	1	0	1	1	1	1	0	1	1	0	1	0	1	1	79%
																				(15/19)
An appropriate justification is provided for modelling	1	0	0	0	1	0	NA	1	1	1	1	0	1	0	1	1	NA	1	NA	63%
																				(10/16)
Modelling hypotheses, assumptions and data sources are presented in a clear and transparent way	0	1	1	0	1	1	NA	1	1	1	1	1	1	1	1	1	NA	1	NA	88%
																				(14/16)
The model has face validity	0	1	1	0	0	0	NA	1	1	1	1	0	1	0	0	0	NA	1	NA	50%
																				(8/16)
Uncertainty surrounding cost-effectiveness/cost-utility is analysed and presented using appropriate statistical techniques	0	0	0	0	0	1	0	1	1	0	1	1	1	0	1	0	0	0	0	37%
																				(7/19)
Future costs are discounted at 3% and future benefits at 1.5%	0	1	1	0	1	1	NA	1	1	1	1	NA	1	1	1	1	NA	1	NA	87%
																				(13/15)

Notes: ‘NA’ = not applicable; ‘1’ = compliant with methodological item; ‘0’ = not compliant with methodological item.

In order to obtain a quantitative summary of compliance with KCE guidelines, a value of ‘1’ was assigned to an item for which the economic evaluation complied with guidelines or for which an adequate justification was provided in case of non-compliance. Items received a value of ‘0’ if the economic evaluation did not comply with guidelines. For each item, the rate of compliance (%) was then calculated, with the denominator reflecting the number of economic evaluations for which the item was applicable (economic evaluations for which the item was not relevant were excluded). 

The assessment of the methodological quality in the standardized reporting template was then compared with the assessment of the economic evaluation by the Drug Reimbursement Committee in the scientific evaluation report. To this effect, any methodological issues raised by the Drug Reimbursement Committee were added or highlighted in a different colour in the standardized reporting template. The response from the pharmaceutical company to the Drug Reimbursement Committee assessment was considered with a view to examining the validity of the issues raised by the Drug Reimbursement Committee.

## Results

### Methodological quality of economic evaluations

Table 1 presents compliance of economic evaluations in Belgian drug reimbursement applications with KCE guidelines [3]. Each methodological item is described in the following sections.

Fifteen out of 19 (79%) economic evaluations were conducted from the health care payer perspective, i.e. the perspective of the National Institute for Health and Disability Insurance (NIHDI) and the patient. The perspective was restricted to that of NIHDI in two economic evaluations and was not reported in two other economic evaluations.

The patient population of all economic evaluations was consistent with the target population identified in the clinical part of the reimbursement application. Six economic evaluations performed subgroup analyses, four of which analysed different subgroups in line with the clinical evidence. In one economic evaluation, subgroup analyses were carried out due to expected differences in costs, but heterogeneity of costs between subgroups was not tested statistically. No justification for subgroup analyses was provided in one economic evaluation.

The comparator in 14 out of 19 (74%) economic evaluations was the most relevant alternative treatment that is most likely to be replaced by the study drug or the treatment without the add-on study drug. No justification for the choice of comparator was given by two economic evaluations and it was not clear whether the comparator reflected the standard of care in Belgium in one economic evaluation. One economic evaluation contrasted the study drug with each possible alternative treatment separately rather than choosing the most relevant alternative treatment as comparator. Finally, one economic evaluation did not compare the study drug (which was a modified-release formulation) with the immediate-release formulation (which was the most relevant alternative treatment in Belgium), even though a clinical trial comparing the two formulations existed. 

Of the four economic evaluations that carried out an indirect comparison between the study drug and comparator, all economic evaluations provided an appropriate justification for the indirect comparison, but none described its limitations or described it in sufficient detail.

Economic evaluations took the form of a cost-minimisation analysis, cost-effectiveness analysis or cost-utility analysis, although an economic evaluation could include multiple analyses. Nine economic evaluations conducted a cost-effectiveness analysis, two of which were based on a single, dominant, patient-relevant clinical outcome (as recommended by KCE guidelines [3]). No justification for the choice of cost-effectiveness analysis was provided in two economic evaluations. Two economic evaluations were cost-minimisation analyses, although the study drug had shown statistically significant superiority over the comparator. In three economic evaluations, cost-effectiveness was based on a secondary outcome instead of a dominant outcome.

Nine out of 19 (47%) economic evaluations complied with KCE guidelines [3] in that they carried out a cost-utility analysis due to the relevance of multiple outcomes and/or quality of life. In the ten other economic evaluations, either no justification was provided for the choice of cost-utility analysis (six economic evaluations) or a cost-utility analysis should have been performed due to the relevance of multiple outcomes and/or quality of life (four economic evaluations), but instead a cost-minimisation analysis and/or a cost-effectiveness analysis were conducted.

All economic evaluations conducted an incremental analysis between study drug and comparator. Although five economic evaluations calculated the incremental cost per quality-adjusted life year gained, they did not report the corresponding incremental cost per life year gained (as recommended by KCE guidelines [3]).

All economic evaluations were based to some extent on data from RCTs or observational studies comparing the study drug with the comparator. These data were usually supplemented by data derived from the literature, expert opinion or both. Although some clinical data suffered from weaknesses, KCE guidelines [3] do not provide detailed guidance on the use of clinical data for the purpose of economic evaluation.

All relevant direct medical costs were considered from the health care payer perspective in 13 out of 19 (68%) economic evaluations. The other six economic evaluations did not include all costs of drugs or all drug-related costs, were restricted to drug costs only, did not calculate all costs of adverse events, or did not encompass all relevant direct medical costs.

In 16 out of 19 (84%) economic evaluations, resource use was measured based on observations or literature. Data sources included pivotal clinical trials, sickness funds, public and private databases, treatment guidelines, literature, a drug’s summary of product characteristics and patient chart review. In 14 of these 16 economic evaluations, resource use data were derived from observations or literature in combination with expert opinion. The source of data on resource use was not reported in three economic evaluations.

Unit cost data were derived from validated Belgian sources as listed in KCE guidelines [3] in 16 out of 19 (84%) economic evaluations. In one economic evaluation, some resource use was valued by means of a simple currency conversion of values found in the international literature and, thus, these values did not reflect Belgian unit costs. One economic evaluation derived some unit costs from the international literature and from Belgian expert opinion. Finally, one economic evaluation did not report the source of data on unit costs.

Seven out of 19 (37%) economic evaluations expressed unit costs in values for the current year and, if applicable, inflated values from previous years to the current year by means of the Belgian health index. Four economic evaluations inflated values by means of other indices such as the general inflation index or the consumer price index. The year in which values were expressed and, if applicable, the method of inflation were not reported in seven economic evaluations. Finally, one economic evaluation extrapolated rather than inflated values from previous years for one cost component and did not report the method of inflation for the other cost components. KCE guidelines [3] recommended that the reference price is used for the comparator when generic drugs exist. Of the nine economic evaluations to which this applied, five complied with the guideline (56%).

Sixteen out of 17 (94%) economic evaluations applied final rather than intermediary outcome measures. According to KCE guidelines [3], life expectancy estimates need to be based on Belgian age-specific life tables and on all-cause mortality (without correction for disease-specific mortality). This guideline was complied with in one out of 13 (8%) economic evaluations to which it applied. In four economic evaluations, life expectancy was not based on Belgian age-specific life tables, but did reflect all-cause mortality. Six economic evaluations drew on a combination of all-cause mortality and disease-specific mortality. Life expectancy estimates reflected international disease-specific mortality in two economic evaluations.

When calculating quality-adjusted life years, health states were described by patients using a generic instrument and valued by the Belgian general public in one out of 15 (7%) economic evaluations for which this methodological item was relevant. In seven economic evaluations, a generic instrument was used to describe health states or utility values were mapped from a disease-specific instrument to a generic instrument. However, utility values related to countries other than Belgium in these economic evaluations. Finally, utility values were taken from the international literature without discussion of transferability to Belgium and without specification of the instrument used to describe health states in seven economic evaluations. 

In 15 out of 19 (79%) economic evaluations, the time horizon reflected the period over which costs and outcomes of study drug and comparator are expected. Two economic evaluations used a short time horizon, whereas a lifetime horizon would have been more appropriate. No justification for the choice of time horizon was provided in two economic evaluations.

Ten out of 16 (63%) economic evaluations provided an appropriate justification for modeling. However, six economic evaluations did not report a justification for modeling. Modeling hypotheses, assumptions and data sources were presented in a clear and transparent way in 14 out of 16 (88%) economic evaluations. Two economic evaluations did not provide sufficient details of modeling data and data sources. Eight out of 16 (50%) economic evaluations provided evidence supporting the face validity of their model. No report of face validity was made in seven economic evaluations. In one economic evaluation, natural progression of the disease appeared to be exaggerated in the model (but lower progression rates were tested in sensitivity analyses).

All but two (89%) economic evaluations carried out sensitivity analyses. Sensitivity analyses were limited to a deterministic sensitivity analysis in one economic evaluation and a probabilistic sensitivity analysis in another economic evaluation. Of the 16 economic evaluations that conducted a deterministic sensitivity analysis, eight did not present results in a Tornado diagram. Of the 16 economic evaluations that performed a probabilistic sensitivity analysis, 14 presented results on a cost-effectiveness plane and 13 in a cost-effectiveness acceptability curve. No report was made of the parameters included in the probabilistic sensitivity analysis and/or the range over which they were varied in four out of 16 economic evaluations.

In line with KCE guidelines [3], 13 out of 15 (87%) economic evaluations discounted future costs at 3% and future benefits at 1.5%. Discounting was not relevant to four economic evaluations with a time horizon equal to or under one year. Two economic evaluations applied discount rates other than those imposed by KCE guidelines [3].

### Quality assessment by Drug Reimbursement Committee

Evaluators of the Drug Reimbursement Committee assess the methodological quality of the economic evaluation in Belgian drug reimbursement applications in the scientific evaluation report. This section evaluates the quality of the assessment by the Drug Reimbursement Committee.

A large variation was observed in the scope and depth of the quality assessment by the Drug Reimbursement Committee. Firstly, the assessment did not always include each of the 11 methodological guidelines issued by the KCE [3]. For instance, the assessment was restricted to one paragraph for one economic evaluation. Secondly, information was not always mentioned under the relevant guideline. For instance, details of modeling were sometimes mentioned under the guideline pertaining to the analytic technique rather than the modeling guideline. Thirdly, the scientific evaluation report generally tended to describe how the economic evaluation was carried out with respect to a guideline instead of assessing compliance with the guideline. For instance, the scientific evaluation report was limited to a summary and did not contain any assessment of quality for two economic evaluations. As a result, many of the cases where an economic evaluation did not comply with KCE guidelines [3] as identified in the previous section (see *Methodological quality of economic evaluations*) were not mentioned in the scientific evaluation report. This also means that the scientific evaluation report did not alert the pharmaceutical company to the case(s) where the economic evaluation did not comply with KCE guidelines [3].

## Discussion

Although methodological guidelines for economic evaluations in drug reimbursement applications may vary from jurisdiction to jurisdiction, results of this study suggest that reimbursement agencies need to assess compliance of submitted economic evaluations with guidelines and need to consult with pharmaceutical industry regarding methodological items from which deviations occur. 

This study has shown that economic evaluations tend to comply with KCE guidelines [3] regarding perspective, target population, subgroup analyses, comparator, use of comparative clinical data and of final outcome measures, calculation of costs, incremental analysis, discounting and time horizon. However, this study also suggests that: a) limitations of indirect comparisons need to be described in more detail in economic evaluations; b) more attention needs to be paid to the choice of an appropriate analytic technique and the justification for that choice; c) unit costs need to be expressed in values for the current year; d) better use could be made of public databases for estimating resource use and costs; e) the estimation and valuation of outcomes (be it life years or quality-adjusted life years) need to be conducted according to methodological guidelines; and f) results of sensitivity analyses need to be presented in the appropriate forms. Although data on modeling were usually presented in a transparent way, the face validity of model inputs and outputs needs to be tested more extensively. Finally, pharmaceutical companies need to ensure that economic evaluations report information on all methodological items addressed by KCE guidelines [3]. Some of these recommendations can be easily addressed, whereas others require that pharmaceutical companies gain a more detailed understanding of the content of KCE guidelines [3] and conduct additional analyses.

Some caution needs to be exercised when assessing the methodological quality of economic evaluations in Belgian drug reimbursement applications. Firstly, no economic evaluation is perfect from a methodological point of view. For instance, a recent study investigated the cost-effectiveness of drugs in Europe based on 231 economic evaluations included in the Tufts Medical Center Cost-Effectiveness Analysis Registry [6]. The methodological quality was assessed by the Registry on a scale from one to seven. The maximum score that any of the included economic evaluations attained was six. Secondly, the assessment of methodological quality used in this study was strict: any deviation from KCE guidelines [3] was categorized as non-compliance. Also, if an economic evaluation did not provide information with respect to a guideline, this was coded as non-compliance. Compliance was coded as a binary variable and did not consider the extent to which an economic evaluation complied with a guideline. Thirdly, the appropriateness of KCE guidelines [3] can be debated as different possible approaches exist for some methodological items. Furthermore, given that KCE did not consider the importance of each of the 11 guidelines, it could be argued that each guideline is equally important and that the importance of non-compliance does not depend on the guideline. This is probably not the case and for example a methodological checklist has been proposed in the literature in which individual items are weighted based on their importance [7].

This study has some limitations. The assessment of methodological quality of economic evaluations in Belgian drug reimbursement applications was carried out by one researcher and not by multiple independent researchers. This approach was chosen because the researcher was involved as an expert in the development of the KCE guidelines [3], has previously carried out assessments of orphan drug reimbursement applications in Belgium [8], and has contributed to the design and conduct of economic evaluations for the purpose of Belgian reimbursement applications. 

It should also be noted that 2 out of 19 economic evaluations in the sample of Belgian drug reimbursement applications were in fact simplistic cost analyses rather than full economic evaluations. However, the response from the firm to the scientific evaluation report included a full economic evaluation for one of these two reimbursement applications. The assessment of methodological quality related to the original submissions.

This study focused on economic evaluations submitted as part of a drug reimbursement application. An assessment of the quality of budget impact analyses submitted as part of a drug reimbursement application fell outside the scope of this study. Also, no official methodological guidelines on budget impact analysis were available during our study period.

This study carried out a quantitative analysis of the methodological quality of economic evaluations included in reimbursement applications, the assessment of their quality by the Belgian Drug Reimbursement Committee and the response to that assessment by the pharmaceutical company. No information was available to conduct a qualitative analysis of how the response by the pharmaceutical company influenced the assessment by the Belgian Drug Reimbursement Committee or how the assessment influenced the decision to (not) reimburse the drug.

Although this study did not explore the association between the methodological quality of economic evaluations and the quality of the assessment by the Drug Reimbursement Committee at the level of individual reimbursement applications, the study did provide evidence of the large variation in the scope and depth of the quality assessment by the Drug Reimbursement Committee. Also, a number of recommendations can be made to improve the quality assessment by the Belgian Drug Reimbursement Committee. There is a need for further education and training in pharmaco-economics for evaluators. Also, more attention needs to be paid to assessing the quality of the economic evaluation in the scientific evaluation report rather than summarizing how the economic evaluation is carried out. Evaluators need to assess all relevant methodological items that are included in KCE guidelines [3]. These steps are needed to ensure that the quality of economic evaluations in drug reimbursement applications is assessed in a uniform and consistent way by the Belgian Drug Reimbursement Committee.

## Conclusions

There is room to enhance the methodological quality of economic evaluations included in Belgian reimbursement applications for Class 1 drugs. Although KCE imposes general guidelines, both pharmaceutical companies and the Drug Reimbursement Committee would benefit from the existence of a more detailed checklist of methodological items (such as the one used in this study) that need to be addressed and reported in an economic evaluation in a drug reimbursement application.
